# Sciatica, of Several Years’ Standing, Cured in Three Weeks, by Replacing a Prolapsed Uterus

**Published:** 1874-10-15

**Authors:** S. J. Avery


### SCIATICA, OF SEVERAL YEARS' STANDING, CURED IN THREE WEEKS, BY REPLACING A PROLAPSED UTERUS.

By Dr. S. J. Avery.

ABOUT the middle of August, 1871, I was called to see Miss T. I found the patient a young woman 21 years of age, of slight figure, a little below medium height, light complexion, and nervous temperament, suffering with pain of the right thigh and leg. The following history of the case I learned from the patient: Some three years before, while employed as teacher in an academy, in the state of New York, she was attacked with severe pains referred to the back and hips, extending down to the thighs. During the following twelve weeks she was confined to her room, and a large part of the time to her bed, being unable to move without assistance. She then began to improve in strength, and was soon after able to take short walks, but was not free from pain or lameness from the first attack to the present time. During this period she had suffered several attacks similar to the first, lasting two or three weeks, confining her as at the first to her room and bed. She had also suffered from the first from dysmenorrhœa.

Upon learning the foregoing history, I informed the mother of the patient, a woman of more than ordinary intelligence, that I suspected uterine difficulty, and probably displacement, as the cause of her daughter's illness. She replied that their "old family physician," in whose judgment and skill they had learned, after an acquaintance of twenty-five years, to place implicit confidence, as also several other medical gentlemen in whose care she had been placed, had never suggested any difficulty of that nature; that it was the desire of herself and daughter that electricity be tried, as that had been highly recommended. Acceding to their wishes, I applied the interrupted current, using one of Kidder's batteries, over the course of the sciatic nerve, twice a week for nearly three weeks, the patient taking bark and iron as a tonic.

Under this treatment she seemed to improve, and expressed a hope that she was really recovering her health.

About this time I was called in haste, the messenger saying that Miss T. was in spasms.

On my arrival I found her suffering from excessive dysmenorrhœa, which was soon relieved by the use of small doses of chloral hydrate, combined with bromide of potassi. One week after, upon digital examination, I found the vulva, beside being somewhat swollen, very tender and sensitive to the touch, the os resting upon the unruptured hymen, which was apparently the only obstacle to complete procidentia.

I succeeded, with some difficulty on account of the swollen and tender condition of the parts, in placing the uterus in its normal position, and left the patient with directions to rest in the horizontal position as much as possible, promising to return in three days.

On the third day following I found the patient much improved. She stated that she had been free from pain since my last visit. The day before being Sunday, she had walked to and from church twice, a distance of nearly half a mile, without any inconvenience, and felt better than she had before since the first attack. I again replaced the uterus, which had fallen about one-third the length of the vagina, and left, promising to call again in one week.

I visited her twice after this, when I found the displacement so slight that further treatment was unnecessary. She took no medicine while receiving local treatment.

She now returned to her home in the east.

Fourteen months after I received a letter from her stating that she continued to improve, had gained 16 lbs. in weight, and that she considered her health established. The points of interest in this case are: First, the importance of a correct diagnosis of the causes of back ache and sciatica in the female. Second, the *probability* of immediate relief in all cases of coincident retroversion, by adjusting the womb. Third, the possibility of the cure being permanent, even without an instrument, care being taken to convert the *retro*version into its antipodes—an *ante*version—in order to secure the antagonism of the normal intestinal pressure.

